# Public-private partnerships as driving forces in the quest for innovative medicines

**DOI:** 10.1186/2001-1326-2-2

**Published:** 2013-01-15

**Authors:** Michel Goldman, Carolyn Compton, Barbara B Mittleman

**Affiliations:** 1 Innovative Medicines Initiative, TO 56, Office 6/4, Brussels, 1049, Belgium; 2 Critical Path Institute, 1730 East River Road, Tucson, AZ, 85718, USA; 3 National Institutes of Health Program on Public-Private Partnerships, 9000 Rockville Pike, Bethesda, Maryland, 20892, USA

**Keywords:** Drug development, Drug efficacy, Drug safety, Pre-competitive research, Knowledge management, Public-private partnership, Data sharing, Regulators, Standards

## Abstract

**Background:**

Despite progress in translational research, we are still falling short in developing the innovative medicines required to address major public health needs. Furthermore, the failure rate, time, and cost required for registration of a new drug are pushing the economics of the industry to the breaking point. New models of drug development based on collaborative endeavours are badly needed to improve this dire situation.

**Findings:**

In 2004, the US Food and Drug Administration (FDA) introduced the Critical Path Initiative with the intent of modernizing drug development by implementing public-private partnerships (PPP) to share data, expertise, and resources. In response to FDA’s initiative, in the following year the non-profit Critical Path Institute (C-Path) was formed. At the same time, the National Institutes of Health (NIH) Public-Private Partnership program was established. In Europe, the Innovative Medicines Initiative (IMI) supported jointly by the European Union and the European Federation of Pharmaceutical Industries and Associations was launched in 2008. These independent efforts have a common long-term objective, namely to facilitate the emergence of innovative medicines by developing new tools for drug discovery, new indicators for drug efficacy or safety, and new approaches for patient stratification. Herein, we present evidence that PPP already exert a positive impact on the drug development process.

**Conclusions:**

Public-private partnerships represent attractive means to leverage resources dispersed across industry, academia, and voluntary health organizations in order to address multiple challenges of drug development in an era of constrained resources and increased regulatory pressure.

## Overcoming hurdles through pre-competitive research

There are several causes of the insufficient productivity of the current drug development process, including but not limited to: [1] the insufficient integration of the results of academic research into the research and development strategy of pharmaceutical companies, [2] the insufficient interest in academic institutions in regulatory science, methods standardization and concrete applications of basic discoveries, [3] the lack of established criteria to define acceptable risk-benefit ratio for drug approval by regulatory authorities, and [4] economic considerations making the development of certain drugs unattractive from an industry standpoint. PPP can help to overcome some of these hurdles by bringing together pharmaceutical companies, academic institutions, science and regulatory agencies, biotechnology firms, patient advocacy associations, and sometimes other industries dedicated to medical devices, telecommunications and information technology. Other stakeholders in healthcare ecosystem may also be included such as representatives of private and public payers. Several PPP were established on this basis during recent years. Herein, we present common features of 3 of them: the C-Path, the NIH Public-Private Partnership program and the IMI.

The Critical Path Initiative was launched by the FDA in 2004 to drive innovation in the scientific processes through which medical products are developed, evaluated, and manufactured [1]. The C-Path Institute was created as an Arizona-based non-profit body to support this initiative by fostering collaborations between industry, academia and regulators. Funding sources are varied and include grant funding from the FDA, fees from participating member organizations, funding from private foundations, and philanthropic donations, and budgets are determined by the scientific agendas of individual consortia.

The NIH Public-Private Partnership program was initiated to develop a coherent approach and advisory/operational support for a wide range of engagements with various non-governmental organizations (industry, foundations, advocacy organizations, etc.) in the setting of complex, often multi-sector/multi-party, arrangements.

The IMI was launched in 2008 following the establishment of a European technology platform for innovative medicines [2]. With a budget of € 2 billion, IMI is a public-private partnership between the European Union and the European Federation of Pharmaceutical Industries and Associations, aiming at boosting the competitiveness of the pharmaceutical sector across Europe by supporting collaborative networks between the key stakeholders in healthcare [3].

A key characteristic of these PPP is their focus on pre-competitive research themes that are outside the traditional field where pharmaceutical firms compete scientifically and economically [4]. A major area of pre-competitive research is drug safety assessment since the development of new approaches to predict potential side effects is of paramount importance to reduce late-stage drug failures, a shared concern for patients, industry and regulatory authorities alike. This is a key objective of the Predictive Safety Testing Consortium (PSTC) launched by the C-Path and of the SAFE-T consortium launched by the IMI. The FDA, through their recent guidance document for drug development tools, has laid out a path for the qualification of biomarkers for use in specific contexts and for defined purposes, one of which is toxicity detection and monitoring. Operating with advice from FDA scientists, the PSTC consortium of 18 pharmaceutical companies identified 7 biomarkers of drug-induced kidney injury [5], which were then formally qualified for preclinical use by the FDA, the European Medicines Agency (EMA), and the Pharmaceuticals and Medical Devices Agency (PMDA) in Japan. In addition, consortia supported by C-Path, the Foundation for FNIH and the IMI are currently conducting trials to qualify these biomarkers for use in the clinical stages of drug development. Pharmaceutical companies have recently reported to the FDA on the extensive use of these qualified preclinical renal biomarkers in their drug development programs either to reassess the risk associated with promising compounds which were previously eliminated from their development pipeline or to assess novel drug candidates. Another example of PPP contributions in this area is provided by the *in silico* models developed by the IMI consortium eTox to facilitate detection of drug safety signals through data mining or by mimicking the action of a drug on a target organ, i.e. the heart [6].

Interestingly, PPP can also accelerate the exploitation of preclinical models to get preliminary assessment of therapeutic activity. One example is the beta-cell line generated by the IMI consortium Imidia [7], the first fully functional human beta cell line suitable for drug research, which is commercially developed by a small enterprise and used by 3 pharmaceutical firms developing anti-diabetic medicines (http://www.imidia.org).

### A critical role for regulatory authorities

In order to ensure that the new tools developed by PPP can be efficiently used in drug development, they are discussed with regulatory authorities at an early stage. This dialog is obviously facilitated by the neutral platform represented by the PPP [8]. Pre-competitive biomarkers, animal models, and clinical outcome assessments have been termed “drug development tools” (DDT) by the FDA, which established an official process, termed “qualification”, for review and confirmation of their validity for a given context of use. C-Path orchestrates the development of DDT through the sharing of data and expertise and consensus building among participating scientists from industry and academia with FDA participation and iterative feedback. The process culminates in a formal application to FDA for official qualification of the DDT for a given use in product development. Similarly, NIH’s involvement with PPP such as the Biomarkers Consortium provides the synergy between basic and clinical scientists, regulators and industry to facilitate the development and qualification of biomarker tools. Qualified DDT then become open tools and standards for the scientific community which, in turn, may be assured both of the scientific rigor under which they were developed and of the FDA’s understanding and acceptance of their validity.

### Tackling knowledge fragmentation

Knowledge fragmentation is a major hurdle in drug development, and it is therefore not surprising that significant achievements of PPP resulted from pooling and sharing of data from multiple sources. In the US, the C-Path’s Coalition Against Major Diseases (CAMD), in collaboration with the Clinical Data Interchange Standard Consortium, published the first data standard for Alzheimer’s by pooling data on more than 6,000 Alzheimer’s patients who had been included in 21 clinical trials (http://www.c-path.org/CAMD.cfm). By combining these data with those from the Alzheimer’s Disease Neuroimaging Initiative database (a public-private effort of NIH with support from pharmaceutical companies (http://www.adni-info.org) and data from peer-reviewed literature, an Alzheimer’s quantitative disease progression model has been elaborated and submitted for regulatory review. Such models are invaluable in improving the design of therapeutic clinical trials for Alzheimer’s disease. CAMD, through the use of aggregated datasets resulting from data pooling across stakeholders, also is engaged in the qualification of biomarkers to identify individuals with episodic memory loss that have a high likelihood for conversion to Alzheimer’s dementia. One such biomarker, hippocampal volume, was recently qualified by EMA for patient enrichment in clinical trials of novel therapies for Alzheimer’s disease. (http://www.ema.europa.eu/docs/en_GB/document_library/Regulatory_and_procedural_guideline/2011/12/WC500118737.pdf). Now all companies have a tool to exclude those patients suffering from other, non-Alzheimer forms of dementia from undergoing treatment with drugs unlikely to provide a benefit.

On the European side, striking results were obtained by the IMI consortium Newmeds, which analyzed data on more than 20,000 schizophrenia patients enrolled in studies conducted by 5 pharmaceutical companies and the National Institute of Mental Health. The study provided evidence that clinical trials for this condition could be simplified by reducing the duration of observation and the number of patients included (http://www.newmeds-europe.com). Furthermore, IMI launched several projects aimed at developing innovative knowledge management tools to support drug discovery and development [3].

### Extending the boundaries of the pre-competitive space

Interestingly, the boundaries of the pre-competitive space have been extended recently by pharmaceutical companies to include clinical studies aimed at establishing to proof-of-concept for the action of lead compounds and even late phase pre-registration trials in areas where incentives are clearly needed to stimulate the interest of industry. A striking example is the program on antimicrobial resistance recently launched by the IMI to reinvigorate the development of novel antibiotics in Europe. Under this ambitious program with a global budget that could reach €500 million, the discovery of new antibiotics for multi-resistant Gram-negative pathogens will be promoted by supporting research on the penetration and transport of drugs across the bacterial wall and early phase clinical trials with lead compounds, while the approval of new products for multi-resistant *Staphylococcus aureus* infections will be accelerated by supporting late-phase pre-registration trials conducted by a dedicated network of clinical investigators (http://www.imi.europa.eu).

### Concluding remarks

Thus far, the experience gained by PPP provides evidence that collaboration between large pharmaceutical industries, government agencies, academic teams, and biotechnology companies can result in significant advances for the development of innovative drugs. Working collaboratively represents a significant change in the business models of large pharmaceutical firms that has emerged from the challenging economic and regulatory forces faced by the healthcare system. These same forces offer unique opportunities to foster pre-competitive collaboration through PPP. Although the first lessons learned from PPP in US and Europe are encouraging, we should not underestimate the significant challenges they are facing, including the management of intellectual property rights and the competition with Asian and Pacific countries. In our view, the long-term impact of PPP on regulatory guidelines will be the best indicator of their ability to achieve the ultimate objective, namely to align the interests of industry and society for the benefit of patients.

## Authors’ contributions

MG, prepared the first draft and coordinated the contributions to the final manuscript, CC, reviewed the draft and prepared the sections related to the Critical Path Institute, BM, reviewed the draft and prepared the sections related to the Program on Public-Private Partnerships of the NIH. All authors read and approved the final manuscript.

## Authors’ information

Michel Goldman is Executive Director of the Innovative Medicines Initiative. Carolyn Compton is President and CEO of the Critical Path Institute; Barbara Mittleman is Director of the Program on Public-Private Partnerships of the NIH.

## Disclaimer

The opinions expressed in this article do not necessarily reflect the positions and opinions of the institutions with which the authors are affiliated.
